# ETI signaling nodes are involved in resistance of Hawaii 7996 to *Ralstonia solanacearum-induced* bacterial wilt disease in tomato

**DOI:** 10.1080/15592324.2023.2194747

**Published:** 2023-03-30

**Authors:** Ai Xu, Lan Wei, Jingjing Ke, Chengfeng Peng, Pengyue Li, Changqiu Fan, Xiao Yu, Bo Li

**Affiliations:** a State Key Laboratory of Agricultural Microbiology, Huazhong Agricultural University, Wuhan, Hubei, China; b The Provincial Key Lab of Plant Pathology of Hubei Province, College of Plant Science and Technology, Huazhong Agricultural University, Wuhan, Hubei, China; c Hubei Hongshan Laboratory, Wuhan, Hubei, China

**Keywords:** *Ralstonia solanacearum*, Hawaii 7996, resistance mechanism, effector-triggered immunity

## Abstract

Bacterial wilt caused by the soil-borne pathogen *Ralstonia solanacearum* is a destructive disease of tomato. Tomato cultivar Hawaii 7996 is well-known for its stable resistance against *R. solanacearum*. However, the resistance mechanism of Hawaii 7996 has not yet been revealed. Here, we showed that Hawaii 7996 activated root cell death response and exhibited stronger defense gene induction than the susceptible cultivar Moneymaker after *R. solanacearum* GMI1000 infection. By employing virus-induced gene silencing (VIGS) and CRISPR/Cas9 technologies, we found that *SlNRG1*-silenced and *SlADR1*-silenced/knockout mutant tomato partially or completely lost resistance to bacterial wilt, indicating that helper NLRs SlADR1 and SlNRG1, the key nodes of effector-triggered immunity (ETI) pathways, are required for Hawaii 7996 resistance. In addition, while SlNDR1 was dispensable for the resistance of Hawaii 7996 to *R. solanacearum*, SlEDS1, SlSAG101a*/b*, and SlPAD4 were essential for the immune signaling pathways in Hawaii 7996. Overall, our results suggested that robust resistance of Hawaii 7996 to *R. solanacearum* relied on the involvement of multiple conserved key nodes of the ETI signaling pathways. This study sheds light on the molecular mechanisms underlying tomato resistance to *R. solanacearum* and will accelerate the breeding of tomatoes resilient to diseases.

## Introduction

*Ralstonia solanacearum* as a soil-borne pathogen, can cause bacterial wilt in tomato, leading to huge economic losses worldwide every year.^1^ *R. solanacearum* first colonizes the root surface and enters roots through natural openings or wounds. A multitude of plant cell-wall-degrading enzymes, as well as exopolysaccharides (EPS) and dozens of type III effectors, are subsequently secreted by the pathogen, which promote its proliferation into stem xylem vessels. The aerial parts of tomato plants are rapidly occupied by *R. solanacearum*, resulting in wilting and death.^2^ Despite the application of diverse chemical or biological management strategies to control bacterial wilt of tomato, they appear to be less effective.^3^ Therefore, disease resistance breeding is widely regarded as the best approach for controlling this devastating disease.^4^

*Solanum lycopersicum* cultivar Hawaii 7996 is a stable resistant resource against diverse *R. solanacearum* strains under different environmental conditions.^5^ Previous studies have shown that restricted shoot colonization of *R. solanacearum* in resistant tomatoes is attributed to limited root invasion,^6^ which prevents the migration of the bacteria from the root collar to the midstem.^7^ Grafting with Hawaii 7996 as the rootstock is one of the most efficient methods to constrain *R. solanacearum* invasion and hinder its spread into the scion.^8^ The comparison of the transcriptional responses has revealed that resistant Hawaii 7996 roots initiated a stronger and earlier activation of defense-related genes than susceptible West Virginia 700 tomato roots upon inoculation with *R. solanacearum*.^9^ The major quantitative trait loci (QTLs) for Hawaii 7996 resistance have been detected on chromosomes 12 and 6 (Bwr-12 and Bwr-6) from the recombinant inbred lines of Hawaii 7996 and West Virginia 700.^10,11^ The analysis of genome-wide single-nucleotide polymorphisms (SNPs) revealed that 4 genes encoding putative leucine-rich repeat (LRR) receptor-like proteins were tightly linked to the chromosome 12.^12^ However, the responsible genes contributing to Hawaii 7996 resistance have not been identified successfully yet.

Plants have evolved a layered innate immune system to fend off potential pathogens. On the one hand, cell-surface localized pattern recognition receptors (PRRs) can recognize pathogen-related molecular patterns (PAMPs) or damage-related molecular patterns (DAMPs) to mount pattern-triggered immunity (PTI).^13^ Receptor-like kinases (RLKs) or receptor-like proteins (RLPs) are representative members of plant PRRs.^14,15^ A typical plant RLK contains an extracellular ectodomain, a single-pass transmembrane domain, and a cytoplasmic kinase domain, whereas an RLP largely resembles an RLK but lacks a cytoplasmic kinase domain.^16^ Leucine-rich repeat (LRR) domain-containing RLKs and RLPs consist of the largest subfamily of plant PRR receptors. To transduce immune signals perceived by the ectodomains, LRR-RLP constitutively interacts with the LRR-RLK suppressor BIR1 (SOBIR1) through their transmembrane domains. In some cases, BRI1-ASSOCIATED KINASE-1 (BAK1) has been identified as a co-receptor indispensable for LRR-type PRR-mediated immunity.^17^ On the other hand, plants employ intracellular nucleotide-binding leucine-rich repeat (NLR) proteins to detect pathogen effectors and activate effector-triggered immunity (ETI).^18,19^ The NLR superfamily mainly consists of three subgroups: toll/interleukin-1 receptor NLRs (TNLs), helical coiled-coil NLRs (CNLs), and RPW8-like coiled-coil domain NLRs (RNLs).^20,21^ ETI usually results in strong plant defense responses, or even local programmed cell death, to restrict pathogen colonization, which is known as the hypersensitive response (HR).

In *Arabidopsis*, Non-race-specific Disease Resistance 1 (NDR1) has been identified as a critical component in CNL signaling pathway.^22^ ENHANCED DISEASE SUSCEPTIBILITY1 (EDS1), which is a lipase-like protein, is required for TNL-mediated resistance and can activate transcriptional defense response and HR.^23^ In addition, another two EDS1 family proteins PHYTOALEXIN DEFICIENT 4 (PAD4) and SENESCENCE-ASSOCIATED GENE101 (SAG101) participate in basal resistance and ETI.^24,25^ Helper NLRs, such as ACTIVATED DISEASE RESISTANCE 1 (ADR1)-related and N REQUIREMENT GENE 1 (NRG1)-related RNLs, play a critical role in TNL signal network in plants. Recent studies have indicated that NRG1/EDS1/SAG101 and ADR1/EDS1/PAD4 are two distinct modules which cooperate to trigger immune signaling pathways.^26^ Moreover, the mutual potentiation between PTI and ETI has been well documented, and LRR-RLP can mediate PTI responses in a EDS1/PAD4/ADR1 module-dependent manner.^21^ Nevertheless, less is known about the potential effects of different immune pathways on the resistance of Hawaii 7996 against *R. solanacearum*.

In this study, we investigated the resistance mechanism of Hawaii 7996 against *R. solanacearum*. Using VIGS technology, we demonstrated that the immune signaling pathways depended on EDS1-PAD4-ADR1 and EDS1-SAG101-NRG1 nodules in the resistant cultivar Hawaii 7996. This study advances our understanding of molecular mechanism underlying resistance of Hawaii 7996 to *R. solanacearum* and offers new strategy for breeding disease-resistant crops against this devastating pathogen.

## Materials and methods

### Plant materials and growth conditions

Hawaii 7996 and Moneymaker (*Solanum lycopersicum*) were grown indoors at 22°C, 75 μE·M^−2^ s^−1^ (T5 LED Tube Lights, 4000K), and 45% relative humidity with 12 h light/12 h dark photoperiod. Tomato seeds were germinated in 1/2 Murashige and Skoog (MS) plates containing 1% (w/v) sucrose and 0.8% (w/v) agar and grown for 14 days.

### Bacterial strains

*Ralstonia solanacearum* strain GMI1000 was grown on CPG solid medium (1 g casamino acid, 10 g peptone, and 5 g glucose, 20 g agar, and 1 L water) at 28°C for 2 days and cultured in CPG liquid medium for 12 hours. *Agrobacterium tumefaciens* strain GV3101 carrying different constructs was incubated in LB medium (10 g tryptone, 5 g yeast extract, and 10 g NaCl, and 1 L water) with 25 μg mL^−1^ gentamicin and respective antibiotics at 28°C for 12 hours.

### Plasmid construction

The cDNA of Hawaii 7996 was used as a template to amplify the coding sequences of *SlADR1*, *SlNRG1*, *SlEDS1*, *SlNDR1*, *SlPAD4*, *SlSAG101a*, *SlSAG101b*, *SlBAK1a*, *SlBAK1b*, *SlSOBIR1a*, and *SlSOBIR1b* with the primers listed in Table S1. The amplified fragments were cloned into the *pYL156* vector.

### CRIPSR/Cas9 gene editing

The CRISPR/Cas9 binary vectors (pTX) were derived from *pBin19*, and the target sequence and Cas9 of CRISPR/Cas9 were driven by the tomato *U6* promoter and *2 × 35S* promoter, respectively. The recombinant *pTX* vector was designed to produce the deletions of *SlADR1* coding sequence using one sgRNA alongside Cas9 endonuclease gene. The sgRNAs used in this study were presented in Table S1). By *pTXCRISPR/Cas9-SlADR1* plasmid was transformed into Hawaii 7996 using the *Agrobacterium*-mediated transformation method. The transgenic tomato lines were selected based on their kanamycin resistance. Positive detection of each plant was conducted through PCR for verifying indel mutations on the left of sgRNA (Table S1).

### Bacterial inoculation assays

The 20 mL bacterial suspension with OD_600_ of 0.1 was poured onto the wounded roots of 4-week-old tomato plants for *R. solanacearum* soil-drenching inoculation. Visual disease symptoms were scored ranging from 0 to 4 (0, no wilting; 1, 1%-25% wilting; 2, 26%-50% wilting; 3, 51%-75% wilting; and 4, 76%-100% wilting). Bacterial populations were detected at 3-day post inoculation (dpi). Specifically, 1-cm-long tissues above stem base from six independent plants were soaked in 1 mL ddH_2_O after grounding, and diluted at the ratio of 1:10. Afterward, 10 μL bacterial suspension was spread on solid CPG medium and the colony forming units were counted after 2 days incubation at 28°C.

### Evans blue staining

The roots were detached, and completely submerged in a 0.25% (w/v) Evans blue solution (Yuanye, Cat.S19046) for 3 minutes (min). Subsequently, the roots were washed with destaining solution (ethanol: acetic acid: glycerin = 3:1:1) three times (6 h per time) in an 80 rpm horizontal shaker.

### RNA isolation, reverse transcription (RT), and quantitative PCR (qPCR) analysis

Using Trizol reagent from TIANGEN (Cat. DP424), total RNA was extracted from root tissues obtained from 2-week-old Moneymaker and Hawaii 7996 plants grown on 1/2 MS medium or obtained from 4-week-old Hawaii 7996 plants grown in soil. The cDNA was synthesized using reverse transcript kit. Total RNA was treated with MonScript^TM^ dsDNase for 2 min at 37°C, and then the first-strand cDNA was synthesized at 50°C for 30 min (Monad, Cat. MR05201). qPCR analysis was conducted using SYBR green Supermix (Monad, Cat. MQ00401S) with the gene-specific primers (Table S1) on the Analytik-jena qTOWER 3 System following standard protocols. The expression of each gene was normalized to the expression of *SlACTIN* (*Solyc11g005330.1*). qPCR was performed with 1 μL cDNA reaction system with 32 cycles of 94°C for 30 s, 55°C for 30 s, and 72°C for 50 s (Table S1). The melting curve was plotted from 65°C to 95°C (Figure S1, S2, S4 and S5).

### Virus-induced gene silencing in Hawaii 7996

*Agrobacterium tumefaciens* strain GV3101 carrying the tobacco rattle virus (TRV) constructs was cultured for the infiltration of Hawaii 7996. TRV-RNA1 cultures were mixed with equal volume of TRV-RNA2 cultures for silencing target genes. TRV-RNA2 vectors included TRV-green fluorescent protein (*GFP*), TRV-phytoene desaturase (*PDS*), TRV*-SlADR1*, TRV*-SlNRG1*, TRV*-SlEDS1*, TRV*-SlNDR1*, TRV*-SlSAG101a/b*, TRV*-SlPAD4*, TRV*-SlADR1/NRG1*, TRV*-SlBAK1a*, *SlBAK1b*, TRV*-SlBAK1a/b*, TRV*-SlSOBIR1a*, TRV*-SlSOBIR1b*, and TRV*-SlSOBIR1a/b*. Two-week-old Hawaii 7996 plants were infiltrated with *A. tumefaciens* cultures carrying the TRV constructs, and the silencing efficiency was assessed through the leaf chlorosis phenotype of TRV-*SlPDS* silencing. The infiltrated plants were grown indoors at 22°C with a 12 h light and 12 h dark photoperiod for 4 weeks, followed by inoculation assays.

## Results

### ETI-related cell death is involved in Hawaii 7996-mediated resistance to R. solanacearum

**Figure 1.**
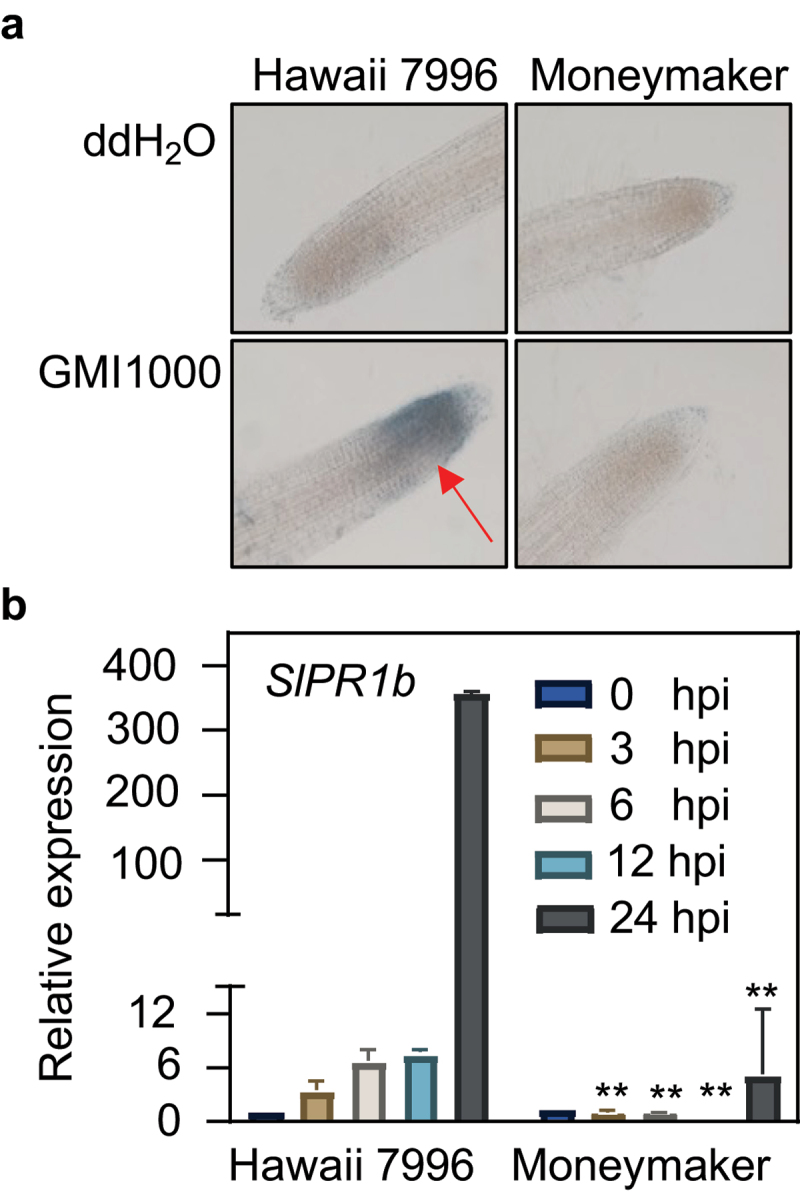
Immune responses in resistant and susceptible tomato varieties to *Ralstonia solanacearum* infection. (a) GMI1000 infection triggers cell death in Hawaii 7996 roots but not in Moneymaker. Evans blue staining was performed with Hawaii 7996 and Moneymaker roots 48 hpi. The cell death area is indicated with red arrows. The above experiments were repeated three times with similar results. (b) The induction of immune-related *SlPR1b* in Hawaii 7996 and Moneymaker roots post GMI1000 infection. Asterisk means significant difference (Student’s one-tailed t-test, * *p* < 0.05, ** *p* < 0.01). Error bars represent ±SD (*n* = 3 technical replicates from one biological assay).

Since Hawaii 7996-mediated resistance to bacterial wilt displayed pathogen strain specificity, we first examined whether HR-like cell death could occur during *R. solanacearum* GMI1000 infection. *R. solanacearum* bacterial infection assay was performed on two-week-old Hawaii 7996 seedlings grown in 1/2 MS plates with the susceptible cultivar Moneymaker as a control. After 48 hours post inoculation (hpi) Evans blue staining, the roots of Hawaii 7996 plants infected with *R. solanacearum* GMI1000 showed obvious cell death, but Moneymaker plant roots did not (Figure 1a). We further investigated the expression of ETI-related maker gene *SlPR1b* (*Pathogenesis-related protein 1b*) in tomato plant roots infected with GMI1000 and detected higher expression level of *SlPR1b* in Hawaii 7996 than in Moneymaker at 24 hpi (Figure 1b, Figure S1). Taken together, the above data indicated that Hawaii 7996 was resistant to *R. solanacearum* GMI1000 and elicited stronger ETI responses such as HR-like cell death than the susceptible cultivar upon *R. solanacearum* infection.

### Helper NLR genes NRG1 and ADR1 are essential for resistance of Hawaii 7996 to R. solanacearum

**Figure 2.**
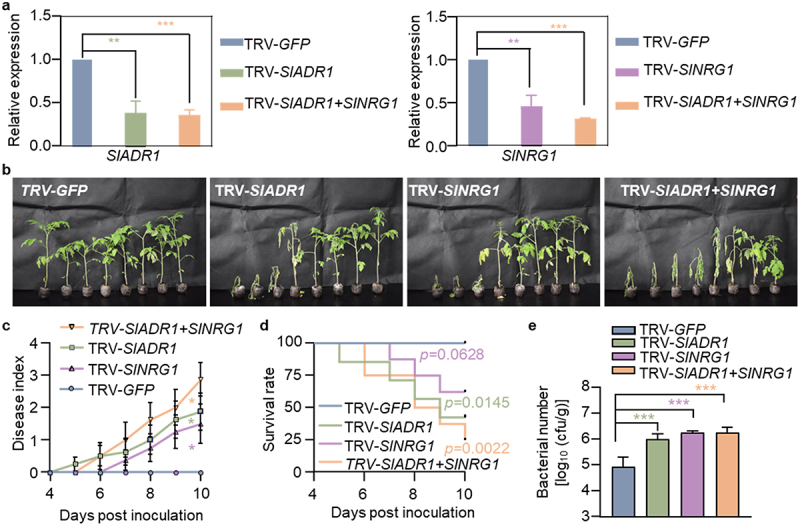
The Helper NLRs SlADR1 and SlNRG1 are critical for tomato resistance to GMI1000 infection. (a) qRT-PCR analysis the induction of *SIADR1* and *SINRG1* in *GFP-*, *SlADR1*-, *SlNRG1*-, or *SlADR1+SlNRG1*-silenced Hawaii 7996 plant roots. (b) Disease symptoms of *GFP*-, *SlADR1*-, *SlNRG1*-, or *SlADR1+SlNRG1*-silenced Hawaii 7996 plants post GMI1000 infection. Soil-drenching inoculation assay were performed with GMI1000 suspension GMI1000 suspension at OD_600_ = 0.1. Pictures of disease symptoms were taken at 9 dpi. (c) Disease index in *GFP-*, *SlADR1*-, *SlNRG1*-, or *SlADR1+SlNRG1*-silenced Hawaii 7996 plants post GMI1000 infection. Error bars represent the ±SD (*n* = 8). (d) The survival ratio of indicated tomato plants post GMI1000 infection. Log-rank (Mantel-Cox) test was used to analyze the corresponding *p* values (*n* = 8). (e) Bacterial growth in the inoculated tomato stems was quantified at 3 dpi from 6 technical replicates. Values represents means ± SD and asterisk indicates a significant difference with control difference (Student’s one-tailed t-test, * p < 0.05, ** *p* < 0.01, *** *p* < 0.001).

NRG1 and ADR1, as helper NLRs, are critical for ETI signaling and cell death induction.^27,28^ To investigate the possible role of *SlNRG1* and *SlADR1* genes in resistance of Hawaii 7996, *Agrobacterium*-mediated TRV-based gene silencing was carried out, and the resistance of *SlNRG1*-, *SlADR1*-, and *SlADR1/SlNRG1*-silenced plants to *R. solanacearum* was evaluated. The RT-qPCR results confirmed a decline in *SlADR1* and *SlNRG1* expression in gene-silenced Hawaii 7996 plants (Figure 2a, Figure S2). Some *SlADR1-* or *SlNRG1-*silenced plants showed clear bacterial wilt symptom, compared to the control TRV-*GFP* plants (Figure 2b). The disease symptom (Figure 2b) and disease index (Figure 2c) suggested that the *SlADR1/SlNRG1-*co-silenced plants were more susceptible to GMI1000 infection than the plants silencing *SlADR1-*or *SlNRG1* individually. After inoculation with GMI1000, the *SlADR1-* or *SlNRG1-*silenced plants exhibited a lower survival rate than the control TRV-*GFP* plants, and *SlADR1*/*SlNRG1-*co-silenced plants displayed the lowest survival rate (Figure 2d). Significantly higher bacterial titers were also counted in tomato stems of gene-silenced plants than those of TRV-*GFP* control plants at 3 dpi (Figure 2e). These results indicated that *SlNRG1* and *SlADR1* are involved in Hawaii 7996 resistance to *R. solanacearum* GMI1000.

**Figure 3.**
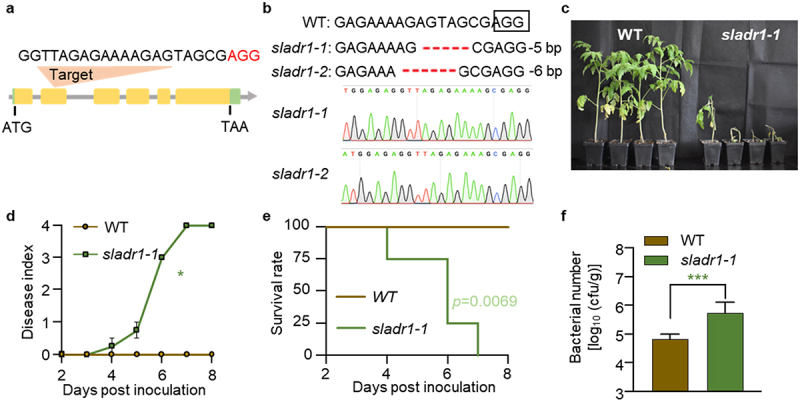
CRISPR/Cas9-induced mutations in the *SlADR1* gene. (a) Schematic illustration of the gRNA target site on the genomic regions of *SIADR1*. Intergenic region and introns are shown as lines; exons are shown as yellow boxes; 3’UTR and 5’UTR are shown as Green boxes. The PAM motifs (NGG) are shown in red. (b) Sequencing analysis of *SIADR1* gene mutation of the *sladr1–1* and *sladr1–2* alleles. The nucleotide deletions are highlighted in red dashed lines and the PAM sequence are boxed. DNA sequencing was shown as chromatographs on the bottom. (c) Disease symptoms of WT and *sladr1–1* mutant plants upon GMI1000 infection. Soil-drenching inoculation assay were performed. Pictures were taken at 7 dpi. (d) Disease index in WT and *sladr1–1* mutant plants upon GMI1000 infection. Error bars represent the ±SD (*n* = 4). (e) The survival ratio of WT and *sladr1–1* mutant plants post GMI1000 infection. Log-rank (Mantel-Cox) test was used to analyze the corresponding *p* values (*n* = 4). (f) Bacterial growth in the inoculated stems of WT and *sladr1–1* mutant plants was quantified at 3 dpi from 8 technical replicates. Values represents means ± SD and asterisk indicates a significant difference with control difference (Student’s one-tailed t-test, * *p* < 0.05, ** *p* < 0.01, *** *p* < 0.001).

To further determine the essential role of *SlADR1* in the pathogen resistance of Hawaii 7996, we generated *sladr1* mutant using the CRISPR/Cas9 gene editing technology. The selected editing site was located at exon 2 of *SlADR1* (Figure 3a). Sequencing analysis of these kanamycin-resistant transgenic plants identified a positive line containing *SlADR1* mutation. The two DNA strands of *sladr1* mutants contained different base deletions with one strand carrying 5 bp (AGTAG) deletion, and the other strand 6 bp (AGAGTA) deletion (Figure 3b). Compared to the WT plants, the *sladr1–1* mutant plants were slightly smaller with fewer leaves after 3 weeks incubation (Figure S3). Next, we examined whether *SlADR1* was required for the resistance of Hawaii 7996 against GMI1000 through bacterial inoculation assay. The results showed that, in contrast to the WT plants, *sladr1–1* mutant plants were more susceptible and developed disease symptoms much faster (Figure 3c–d). The mutant plants completely wilted at 7 dpi, whereas most of WT plants were healthy and showed resistance to GMI1000 infection (Figure 3e). Bacterial titers in *sladr1–1* mutant plants were extraordinarily elevated in comparison to WT plants (Figure 3f). The above results indicated that *sladr1–1* mutant plants completely lost the resistance to *R. solanacearum* GMI1000 infection.

Figure 3b: The box of the PAM sequence has shifted

### Resistance of Hawaii 7996 is dependent on EDS1-related complex

**Figure 4.**
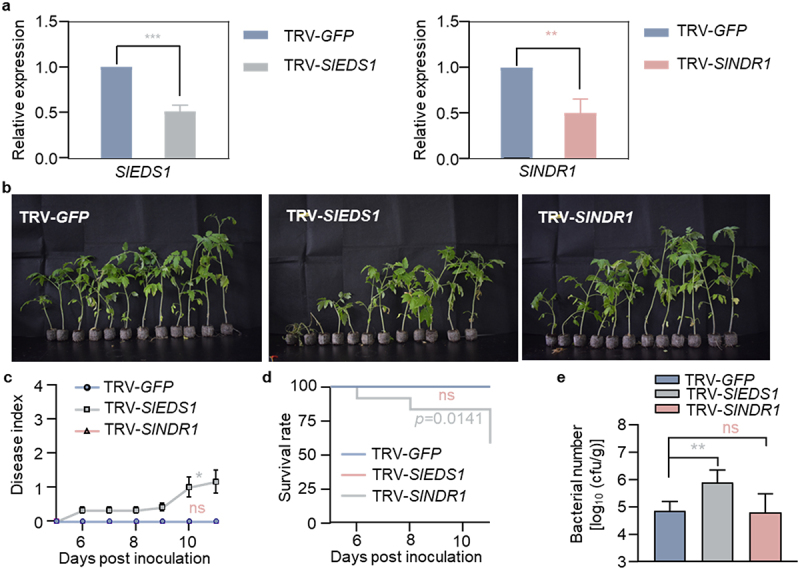
Tomato bacterial wilt resistance depends on EDS1 rather than NDR1. (a) qRT-PCR analysis the induction of *SIEDS1* and *SINDR1* in *GFP-*, *SlEDS1*-, or *SINDR1*-silenced Hawaii 7996 plant roots. (b) Bacterial wilt symptoms of *GFP*-, *SlEDS1*-, and *SlNDR1*-silenced Hawaii 7996 plants post GMI1000 infection. Soil-drenching inoculation assay were performed with GMI1000 suspension at OD_600_ = 0.1 on indicated plants. Pictures of disease symptoms were taken at 11 dpi. (c) Disease index was quantified in *GFP*-, *SlEDS1*-, and *SlNDR1*-silenced plants. Error bars represent ±SD (*n* = 12). (d) The survival ratio of indicated tomato plants post GMI1000 infection. Log-rank (Mantel-Cox) test was used to analyze the corresponding *p* values (*n* = 12). (e) Bacterial growth in the inoculated tomato stems was quantified at 3 dpi. Values represents means ± SD (*n* = 6 individual plants) and asterisk indicates a significant difference with control difference (Student’s one-tailed t-test, * *p* < 0.05, ** *p* < 0.01, *** *p* < 0.001).

In *Arabidopsis*, EDS1-PAD4-ADR1 complex is essential for TNL-induced basal resistance, whereas EDS1-SAG101-NRG1 complex is a critical component for TNL-mediated cell death.^27,29^ Meanwhile, EDS1 and NDR1 are the conserved signaling nodes in TNL and CNL pathways, respectively.^23,30^ To elucidate the roles of EDS1 and NDR1 in resistance of Hawaii 7996 to *R. solanacearum*, leaves of Hawaii 7996 plants were infiltrated with *Agrobacterium* carrying TRV2-*SlEDS1* or TRV2-*SlNDR1*. The results showed that the expression of *SlEDS1* and *SlNDR1* was significantly reduced in corresponding gene-silenced plants (Figure 4a, Figure S4a-S4b). Three weeks after VIGS, the resistance of the gene-silenced plants to *R. solanacearum* GMI1000 was determined. The data showed that *SlEDS1-*silenced plants, but not *SlNDR1*-silenced plants, were more severe in disease symptoms (Figure 4b) and disease index (Figure 4c) when compared to the TRV-*GFP* control plants. Additionally, a relatively lower survival rate was observed in *SlEDS1-*silenced plants than control plants at 11 dpi (Figure 4d). Bacterial titers in stems of tomato plants with TRV-*SlEDS1* were 10 times higher than those of the control plants TRV-*GFP* (Figure 4e). Collectively, these results suggested that *SlEDS1* is required for the resistance of Hawaii 7996 against *R. solanacearum*.

**Figure 5.**
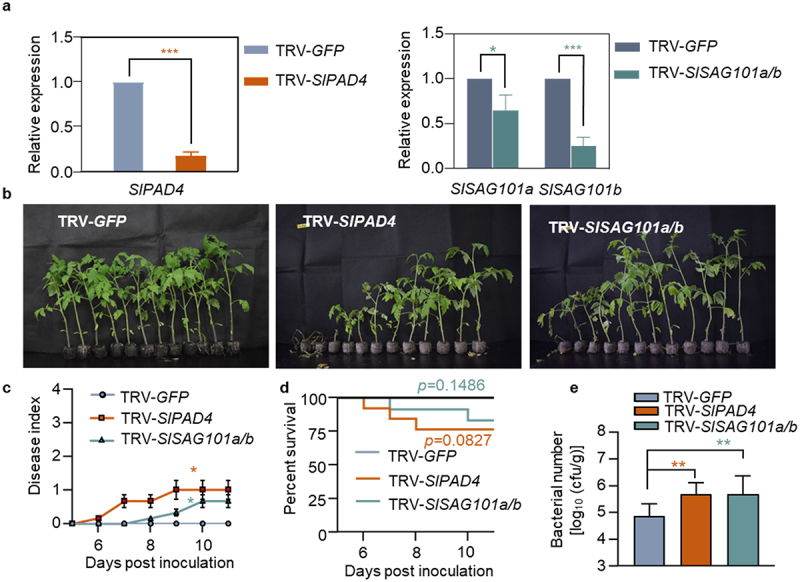
The requirement of tomato PAD4 and SAG101 for bacterial wilt resistance. (a) qRT-PCR analysis the induction of *SIPAD4*, *SlSAG101a* and *SlSAG101b* in *GFP-*, *SlPAD4*-, or *SlSAG101a/b*-silenced Hawaii 7996 plant roots. (b) Bacterial wilt symptoms of *GFP*-, *SlPAD4*-, and *SlSAG101a/b*-silenced Hawaii 7996 plants post GMI1000 (OD_600_ = 0.1) infection. Pictures were taken at 11 dpi. (c) Disease index was quantified in *GFP*-, *SlPAD4*-, and *SlSAG101a/b*-silenced plants. Error bars represent ±SD (*n* = 12). (d) The survival ratio of indicated tomato plants post GMI1000 infection. Log-rank (Mantel-Cox) test was used to analyze the corresponding *p* values (*n* = 12). (e) Bacterial growth in the inoculated tomato stems was quantified at 3 dpi from 6 technical replicates. Values represents means ± SD and asterisk indicates a significant difference with control difference (Student’s one-tailed t-test, * *p* < 0.05, ** *p* < 0.01, *** *p* < 0.001).

To investigate whether the other components in these signaling modules also participate in the resistance of Hawaii 7996, we performed VIGS to silence *SlPAD4* or *SlSAG101a/b* in tomato plants, then challenged the plants with GMI1000. The qPCR results showed that the target genes were specifically silenced without affecting the expression of the close homologues in the EDS1 family (Figure 5a, Figure S4c-S4h). Both *SlPAD4-* and *SlSAG101a/b-*silenced plants exhibited higher susceptibility (Figure 5b) and higher disease index (Figure 5c) than the control plants. Typical bacterial wilt symptoms were observed in TRV*-SlPAD4-* or TRV*-SlSAG101a/b-*silenced plants at 7 dpi (Figure 5d), and bacterial titers in these silenced plants were 6 to 7 folds higher than in TRV-*GFP* plants at 3 dpi (Figure 5e). These results demonstrated that *SIPAD4* and SI*SAG101a/b* were crucial for the pathogen resistance of Hawaii 7996.

### PRR coreceptors contribute to Hawaii 7996 resistance

**Figure 6.**
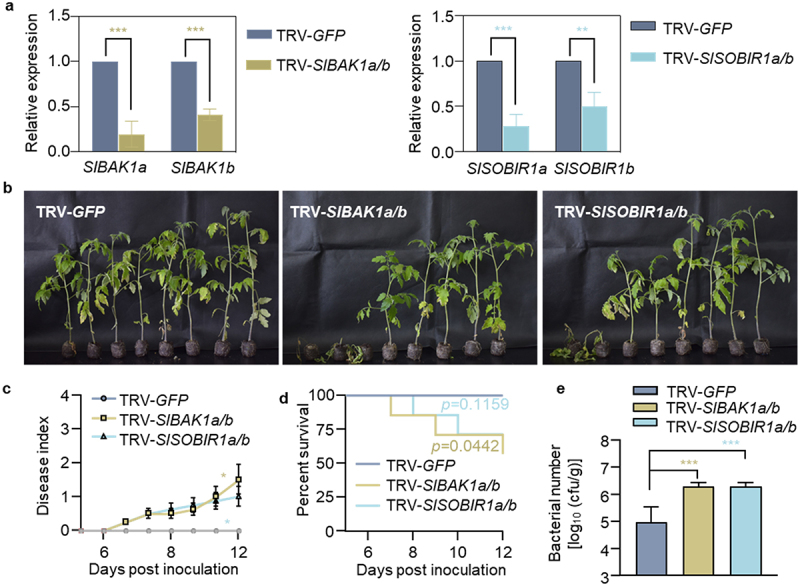
Tomato BAK1 and SOBIR1 partially contribute to bacterial wilt resistance. (a) qRT-PCR analysis the induction of *SlBAK1a, SIBAK1b, SISOBIR1a* and *SISOBIR1b* in *GFP-*, *SlBAK1a/b*-, or *SISOBIR1a/b*-silenced Hawaii 7996 plant roots. (b) Bacterial wilt symptoms in *GFP*-, *SlBAK1a+1b*-, and *SISOBIR1a+1b*-silenced Hawaii 7996 plants post GMI1000 infection. Soil-drenching inoculation assay were performed using GMI1000 suspension at OD_600_ = 0.1. Pictures were taken at 11 dpi. (c) Disease index was quantified in *GFP*-, *SlBAK1a/b*-, and *SISOBIR1a/b*-silenced plants post GMI1000 infection. Error bars represent ±SD (*n* = 8). (d) The survival ratio of indicated tomato plants post GMI1000 infection. Log-rank (Mantel-Cox) test was used to analyze the corresponding *p* values (*n* = 8). (e) Bacterial growth in the inoculated tomato stems was quantified at 3 dpi. Values represents means ±SD (*n* = 6 individual plants) and asterisk indicates a significant difference with control difference (Student’s one-tailed t-test, * *p* < 0.05, ** *p* < 0.01, *** *p* < 0.001). The above experiments were repeated at least three times with similar results.

Considering that EDS1-PAD4-ADR1 is linked to RLP-SOBIR1 receptor complex,^21^ we sought to figure out whether tomato PRR coreceptors BAK1 and SOBIR1 are involved in the resistance of tomato Hawaii 7996. Transient silencing of *SlBAK1a* and *SlBAK1b* encoding *Arabidopsis* BAK1 orthologues, or *SlSOBIR1a* and *SlSOBIR1b* encoding SOBIR1 orthologues were carried out by VIGS, in which these genes were specifically silenced in the corresponding plants (Figure 6a, Figure S5). Then, we investigated the plant resistance against GMI1000 infection. Plants silencing *SlBAK1a*, *SlBAK1b*, *SlSOBIR1a*, or *SlSOBIR1b* individually did not show clear bacterial wilt symptom, compared with the control TRV*-GFP* plants (Figure S6). However, the 25% of *SlBAK1a/b-*silenced plants and 37.5% of *SlSOBIR1a/b-*silenced plants displayed the enhanced susceptibility and faster disease symptoms than the control plants (Figure 6b–c). Meanwhile, we observed a reduced survival rate in TRV*-SlBAK1a/b-* or TRV-*SlSOBIR1a/b-*silenced plants infected with GMI1000 in contrast to TRV*-GFP* plants (Figure 6d). Bacterial titers in the TRV*-SlBAK1a/b-* or TRV*-SlSOBIR1a/b-*silenced plants at 3 dpi were significantly higher than those in the TRV*-GFP* plants (Figure 6e). Our data suggested that tomato *BAK1* and *SOBIR1* are partially required for the resistance of Hawaii 7996 to *R. solanacearum*.

## Discussion

In this study, we compared the immune responses in resistant and susceptible tomato cultivars upon *R. solanacearum* infection, including defense-related gene expression and HR response. We found that induction of *SlPR1b* gene and HR response of the resistant cultivar Hawaii 7996 was faster and stronger than that of susceptible cultivar Moneymaker upon infection with *R. solanacearum*. By deploying VIGS and CRISPR-Cas9 technologies, we found that the resistance of Hawaii 7996 to *R. solanacearum* requires the involvement of multiple key components in the ETI signaling pathway (Figure S7). Our results offer valuable insights in understanding the disease resistance of tomato to bacterial wilt.

Hawaii 7996 has stable and robust resistance against various *R. solanacearum* in tomato and shows a high survival rate, but the corresponding molecular mechanism remains largely elusive. The explorations of the resistance phenotypes at the physiological, metabolism, and transcriptional levels have indicated that Hawaii 7996 can effectively limit the growth, colonization, and EPS production of *R. solanacearum* in vascular tissues.^6^ Hawaii 7996 restricts the *R. solanacearum* infection by limiting bacterial root colonization, vertical movement upwards from root to the stem, the invasion into circular vascular bundles, and radial apoplastic spread in the cortex^8^. Immune gene activation is a rapid and effective defense response for plants against pathogens.^9,31,32^ Cell death or HR occurs in resistance cultivar LS-89 after *R. solanacearum* infection.^33^ Similarly, this study discovered that *R. solanacearum* GMI1000 induced cell death in the roots of Hawaii 7996, which was not observed in Moneymaker. In addition, the induction of immune-related gene *SlPR1b* in Hawaii 7996 was much stronger after GMI1000 inoculation, compared with that in Moneymaker, implying that the strength of ETI was associated with tomato resistance to bacterial wilt.

Further, we revealed that the key node components in the ETI signaling pathway were required for Hawaii 7996 resistance, thereby expanding our knowledge of tomato resistance to *R. solanacearum*. In *Arabidopsis*, TNL pair RRS1/RPS4 can recognize AvrRps4 from *Pseudomonas syringae* and PopP2 from *R. solanacearum*. Helper NLRs was required for multiple sensor NLRs to mediate downstream signaling. For instance, RRS1/RPS4-mediated bacterial resistance requires the participation of RNLs including NRG1A, NRG1B, and ADR1s.^34-36^ However, RRS1/RPS4-mediated HR is dependent on NRG1s but not ADR1s.^27^ In this study, *SlADR1*- and/or *SlNRG1*-silenced Hawaii 7996 plants exhibited impaired resistance to *R. solanacearum*. In addition, our results showed that *sladr1–1* mutant plants completely lost the resistance to GMI1000. These data jointly indicated that helper NLRs ADR1 and NRG1 are involved in Hawaii 7996-mediated resistance to *R. solanacearum*.

NDR1 and EDS1 are conserved critical nodes required for ETI activation. Our data showed that the resistance of Hawaii 7996 was independent of *SlNDR1*, but dependent on *SlEDS1*, which suggested TNLs likely play critical roles in the resistance of Hawaii 7996. Meanwhile, we found that *SlPAD4* and *SlSAG101a/b* are both required to mount effective resistance in Hawaii 7996. In *Arabidopsis*, EDS1-PAD4-ADR1 complex is required for TNL-induced basal resistance, whereas EDS1-SAG101-NRG1 complex is a critical component for TNL-mediated cell death.^27,29^ ADR1 and NRG1 participate in plant immunity as helper NLRs.^34^ Our data indicated that both EDS1-PAD4-ADR1 and EDS1-SAG101-NRG1 modules are strictly required for the resistance of Hawaii 7996 toward *R. solanacearum*. It is noteworthy that PTI and ETI do not operate independently but rather exhibit mutual potentiation, and EDS1-PAD4-ADR1 signaling module is required for RLP-mediated resistance.^37,38^ In this study, we discovered that silencing *SlBAK1a/b* or *SlSOBIR1a/b* resulted in partial loss of resistance of Hawaii 7996 plants to wilt disease, indicating that certain PRR(s) might participate in this resistance. Further, QTLs related to *R. solanacearum* resistance were mapped onto chromosome 12, which contains multiple *RLP* genes.^12^ The exact roles of those candidate RLPs in the resistance against *R. solanacearum* awaits future investigation. The key receptors recognizing *R. solanacearum* in both PTI and ETI pathways in Hawaii 7996 require further exploration. The related findings will aid in understanding the resistance mechanisms of Hawaii 7996 to *R. solanacearum* and provide new insights and solutions to disease resistance breeding of tomato.
